# Can adults with cerebral palsy perform and benefit from ballistic strength training to improve walking outcomes? A mixed methods feasibility study

**DOI:** 10.1186/s13102-021-00382-1

**Published:** 2021-12-18

**Authors:** Beate Eltarvåg Gjesdal, Silje Mæland, Gavin Williams, Mona Kristin Aaslund, Cecilie Brekke Rygh, Kristoffer Toldnes Cumming

**Affiliations:** 1 Department of Health and Function, Western Norway University of Applied Sciences, PO Box 7030, 5020 Bergen, Norway; 2 Department of Global Public Health and Primary Care, Faculty of Medicine, The University of Bergen, Bergen, Norway; 3 Research Unit for General Practice in Bergen, Norwegian Research Centre, NORCE, Bergen, Norway; 4 Department of Physiotherapy, Epworth Hospital, Richmond, Melbourne, VIC Australia; 5 Department of Physiotherapy, The University of Melbourne, Parkville, Melbourne, VIC Australia; 6 Faculty of Health and Welfare and Organisation, Østfold University College, Fredrikstad, Norway

**Keywords:** Gait, Ankle plantarflexors, Resistance training, Interview, Patient reported outcome

## Abstract

**Background:**

Power bursts of hips and ankle plantar flexors are prerequisites to walking propulsion. However, these power bursts are reduced during gait for persons with cerebral palsy (CP) and mainly in the ankle plantar flexors. Hence, task specific training, such as ballistic strength training, is suggested to increase muscle power in walking but not investigated in adults with CP. Therefore, the aim was to investigate if adults with CP could perform and benefit from ballistic strength training to improve walking, evaluated through physical measures and self-reported measures and interviews.

**Methods:**

In this mixed methods feasibility study, eight ambulatory adults (aged 24–56) with spastic CP conducted ballistic strength training on a glideboard targeting the ankle plantarflexors two times a week for eight weeks. The feasibility of the training was assessed through objectives described by Orsmond and Cohn. Before and after the intervention, physical measures (6-Minute Walk Test and the eight-item High-level Mobility Assessment Tool) and self-reported measures (Patient Global Impression of Change, Numeric Pain Rating Scale, Fatigue Impact and Severity Self-Assessment, and Walk-12) were collected. After the intervention, semi-structured interviews explored experiences of this training.

**Results:**

The participants experienced training the ankle plantar flexor as relevant but reported it took about four weeks to coordinate the exercises successfully. Although we observed no changes in the physical performance measures, most participants reported improvements; some felt steadier when standing, walking, and hopping.

**Conclusion:**

This study demonstrated that ballistic strength training was feasible and suitable in adults with CP. However, guidance and a long (4 weeks) familiarization time were reported necessary to master the exercises. Most participants reported self-experienced improvements, although no physical performance measures improved. Thus, prolonged intervention may be required for perceived physical improvements to emerge. Also, other outcome measures sensitive to power output remains to be investigated.

**Supplementary Information:**

The online version contains supplementary material available at 10.1186/s13102-021-00382-1.

## Background

Walking onset is postponed in children with cerebral palsy (CP) due to an injury of the immature brain which may prevent healthy development [1]. There is vast variation in function, from independent individuals to those in need of total care. Hence CP is referred to as an ‘umbrella term’ for all of these individuals [2]. Limited coordination and motor control are often accompanied by disturbances of sensation, perception, cognition, and secondary musculoskeletal problems (like muscle shortening and weakness) [3]. Self-reported factors such as pain, fatigue and joint contracture have been associated with a decline in mobility and balance [3]. Adults with CP report walking deterioration earlier than their peers and the more severely affected report deterioration at the youngest age [4].

Muscle weakness affects joint control during gait [5], and although resistance training improves muscle weakness in CP, walking has not been shown to improved [6]. In a focused review, Williams et al. [7] highlighted that strength training to improve walking should be task-specific to the propulsive power bursts in gait (hip flexors, hip extensors and ankle plantar flexors). However, few neurologic rehabilitation studies target these muscles [7]. Therefore, a theoretical framework suggests ballistic strength training as task specific to increase muscle power relevant for walking in neurologic rehabilitation [8]. Ballistic strength training is a type of power training aiming to increase the rate of force production, and preferable since these exercises eliminates the deceleration phase due to its jumping nature. Muscular power is the scalar product of force generation and movement velocity. Hence both traditional strength training and power training has the potential to increase muscular power [9], but light resistance and rapid excursions are suggested preferable when increasing power [9].

Recent literature reveals a growing interest in power training for improving walking in youths and adults with CP, but different terminology and various muscles are targeted. Moreau et al. [10] reported beneficial results of walking speed in youths with CP after high-velocity strength training compared to traditional training targeting knee extensors. Kirk et al. [11] found increased toe-lift in the late swing after explosive resistance training of the ankle dorsiflexors in adults with CP. These studies suggest that focus on power could benefit walking in this population. However, to our knowledge, ballistic strength training of calf muscles (ankle plantar flexors) has not been studied in adults with CP.

All power bursts relevant for walking are reduced in persons with spastic CP, and mainly ankle power [12]. This could be explained by reduced muscle volume [13], plantarflexor weakness [14] and impaired rapid force generation capacity [15, 16]. Two of the neuromuscular contributors to maximal power are the maximal rate of force development (RFD) and coordination [9]. Ankle plantar flexor RFD has been associated with impaired gait function in adults with CP [16]. Ankle plantar flexor ballistic strength training (power training) focus on angular velocity, is suggested to be task-specific to walking [8], and safe and feasible for adults after traumatic brain injury [17], and in children with CP [18]. However, it is unknown whether this training is feasible, or if it improves walking and how it is experienced by adults with CP.

The purpose of this study was to investigate if adults with CP can perform and benefit from ballistic strength training of the ankle plantar flexors to improve walking. This was evaluated through physical measures, self-reported measures, and interviews.

## Methods

### Design

A mixed methods feasibility design yields a broad perspective when exploring a new intervention in adults with CP. Combining qualitative and quantitative data provide the potential for a better understanding of the research problems [19], and including participants' voices is requested by The Norwegian Ministry of Health and Care Services [20]. Qualitative and quantitative data were rigorously collected and analysed following the convergent design. Further, we compared each individual's qualitative and quantitative data to obtain a thorough understanding, and the results were mixed in the discussion [19].

In line with the goal of a feasibility study, we aimed to answer the question, "Can it (ballistic strength training) work (for adults with CP)?". Several single studies are published to guide authors in conducting a feasibility study. Therefore Orsmond and Cohn [21] synthesised recommendations from seven published articles into five overarching objectives for a feasibility study: "(1) *Evaluation of recruitment capability and resulting sample characteristics,* (2) *Evaluation and refinement of data collection procedures and outcome measures,* (3) *Evaluation of acceptability and suitability of intervention and study procedures,* (4) *Evaluation of resources and ability to manage and implement the study and intervention,* (5) *Preliminary evaluation of participant responses to intervention*". Since this study investigated whether they could perform and benefit from the ballistic training, objectives 2–3–5 [21] are used in this study design and structure of this article's discussion.

### Recruiting and inclusion and exclusion criteria

Information about the study was sent to the regional CP Association, which forwarded it to their adult members. Information was also posted on social media with encouragement for others to share, through posters in training centres and at the Regional Hospital. Inclusion criteria were adults with spastic CP, gross motor function classification system (GMFCS) level I–II. The participants also had to complete primary school to make sure they understood the instructions. The key exclusion criterion was lower limb surgery during the last year before the study intervention. We invited potential participants that met all the criteria to visit the laboratory for an information meeting about the study to ask questions prior to start-up.

### Participants

Ten adults with independent walking function enrolled in the study. One withdrew before commencing because participation was too burdensome. Nine participants came to the pre-test, seven of whom were classified as hemiplegic and GMFCS I, and two of whom were classified as diplegic and GMFCS II (Table 1). None of the participants had previous experience with ballistic training.

**Table 1:** Participant characteristics

Case	Sex	Age	BMI	Subtype	GMFCS	Lab exercise completed (total 16)	Home exercise completed (total 4)
P1	F	27	18	Uni	I	15	2
P2	F	51	23	Uni	I	15	3
P3	F	28	27	Uni	I	16	0
P4	M	53	30	Bilat	II	15	1
P5	M	34	28	Bilat	II	13	2
P6	F	24	24	Uni	I	16	2
P7	F	30	38	Uni	I	16	0
P8	M	56	28	Uni	I	16	2

*BMI* body mass index, *GMFCS* gross motor function classification scale

Ethical approval was granted by the Regional Committees for Medical and Health Research Ethics of West Norway (REK-No. 2018-2390) and all volunteers signed a written informed consent before the study intervention. The study was carried out according to the latest revision of the declaration of Helsinki.

### Intervention

The participants conducted three ballistic exercises targeting ankle plantarflexors twice a week for eight weeks in the rehabilitation laboratory at the University College. An additional home exercises was introduced in week five. Optimal dosage is not established in adults with CP, however frequency (2–3 times per week) and duration (8–10 weeks) is suggested as guidelines when improving muscle power in young adults with CP [10]. All supervised training in the lab started with a general warm-up of either indoor cycling or treadmill walking. Three exercises were performed lying on an inclined glideboard supervised by trained personnel (Total Gym RS Encompass PowerTower®): (a) jump squats, (b) single leg hopping on the paretic leg and (c) bounding on alternating legs. We aimed to incorporate task specific training by closely monitoring exercise performance in each training session with a prioritised list of progression rules of skill acquisition, speed, and the range of motion. After midstance, the ankle moves from dorsiflexion (10°) to plantarflexion (20°) [22]. The load was increased once these performance criteria were met. Participants were encouraged to complete as many repetitions as possible within five minutes. If the exercises were not performed with quality, the load was considered too high. A quality check of muscle activation was to palpate the gastrocnemius muscle during the exercise. This is relevant since a commonly observed challenge was that the knee-extensors compensated for the weak ankle plantarflexors. This was a bigger problem at the beginning of the intervention when the participants needed much more feedback. Therefore, breaks were initiated either by the participant or researcher/training supervisor if ankle plantarflexor technique and or coordination deteriorated. The glideboard incline and number of repetitions, and type of assistance for each session, were recorded. For a detailed description of the exercises, see Appendix 2 in Hendrey et al. [17].

At week five a home exercise was introduced to the participants. The exercises targeted the hip flexor power generation, the second most important muscle group for power-burst in forward propulsion relevant for walking [8]. Demonstration of the home exercises and videos explaining the performance were provided to the participants. Moreover, they were asked to report the home training in a training diary. Three exercises were chosen from a progressive list, e.g., knee lift, claw or triplings, and were instructed to perform three sets of 12 repetitions and report them in the training diary. Although the hip, knee and ankle power contributions are relatively similar across walking speeds [23], hip flexor weakness is found to impact gait [24] and this is why we chose to target these. The home exercises were inspired by athlete exercises task-specific to develop hip flexor power in running, with focus on rapid excursions. Which is why we chose to target these. The home exercises were inspired by athlete exercises task-specific to develop hip flexor power in running, focusing rapid excursions.

Demonstration to the home exercises and videos explaining the performance were provided to the participants. Moreover, they were asked to report the home training in a training diary. Three exercises were chosen from a progressive list, e.g., knee lift, claw or triplings, and were instructed to perform three sets of 12 repetitions and report them in the training diary. Although the hip, knee and ankle power contribution are relatively similar across walking speeds [23]; hip flexor weakness is found to impact gait [24] and this is why we chose to target these. The home exercises were inspired by athlete exercises task-specific to develop hip flexor power in running, with focus on rapid excursions.

Other exercise activities besides the intervention, delayed onset muscle soreness, adverse events or other symptoms limiting participation in the exercise were recorded by the personnel each time the participant came to the laboratory. All subjects were asked to continue their regular training routines (Additional file 1: Home-based training and other training).

### Outcome measures

#### Physical measures

*Physical measures* were performed before and after the intervention.

The *six-minute walk test (6MWT)* measured changes in walking capacity. Participants walked along a 30-m-long circuit and every minute, a standard set phrase was given by the investigator. Heart rate (HR) was measured using a waist belt and HR monitor (Polar, M400) and a perceived exertion scale (Borg 1–20) was registered immediately after the test. The 6MWT is reliable in adults with CP; an increase in walking distance of 40 m is considered clinically significant when a practice test is conducted and 56 m when not conducted [25].

The eight-item version of the *High-level Mobility Assessment Tool (HiMAT)* measured high-level mobility (run, hop and skip). HiMAT has been validated for individuals with neurological conditions [26] and the eight-item HiMAT score was used in the analysis [27]. The maximum score is 32 points, with a higher score indicating better performance. Minimal Detectable Change (MDC) is two points [27].

#### Self-reported measures

The *patient global impression of change (PGIC)* scale was used four times during the intervention (weeks 2, 4, 6 and post-test) to rate participants’ current walking function compared to how it was prior to intervention, on a 7-point scale from ‘Very much better’ to ‘Very much worse’. Any measure other than ‘Unchanged’ is meaningful in PGIC.

The *Numeric Pain Rating Scale (NPRS)* was used to report pain intensity four times (pre-test, week 3, week 6 and post-test) on a scale from ‘0 = no pain’ to ‘10 = worst possible pain’. The NPRS is valid in adults with CP [28] and a clinical change of two points has been reported in patients with low back pain [29].

The *Fatigue Impact and Severity Self-Assessment (FISSA)* instrument measured experienced fatigue related to CP twice during the last week (pre-and post-test). The FISSA is validated and reliable for individuals with CP [30]. The minimum score is 31 and the maximum score is 137, with a higher score indicating greater fatigue.

The *Walk-12* instrument was used to investigate any self-perceived change in walking difficulties related to diagnosis after ballistic strength training. Walk-12 is a generic version of the Multiple Sclerosis Walking Scale-12. The Multiple Sclerosis Walking Scale has the potential to evaluate the neurologic conditions impact on walking, monitor change in walking over time and evaluate therapeutic effectiveness [31]. Holland et al. [32] concluded that this version might be suitable for other neurologically disabled persons [32]. Walk-12 was measured two times (pre-and post-test). The maximum score was 60 points, and the minimum score was 12 points; fewer points indicate fewer perceived walking difficulties. Scores were converted to percentages when presenting data. Walk-12 has the potential to give valuable information on how neurologic disease is related to walking difficulties. No validation has been done for this modified version among adults with CP, but among adults with Multiple Sclerosis, a clinically significant change is 22 points, or 53% [33].

#### Qualitative interviews

*Semi-structured individual interviews* were conducted at post-test. Interviews were related to experiences with ballistic training and the overarching question was ‘What are your experiences with the training performed?’, with the specification that we were interested in both positive and negative experiences. The first and second authors conducted the interviews. The interview guide is given in Additional file 1: Interview guide.

### Analysis

Descriptive statistics were used to describe physical performance and self-reported measures. Results were compared to measures of clinical change to determine the necessary change on an outcome measure to exceed variation and errors in the measurement. Adherence was reported as percentage of the prescribed exercise sessions attended.

The interviews were transcribed verbatim and analysed with systematic text condensation (STC), a four-step thematic cross-case analysis approach [34]. The first and second authors conducted the first three steps of STC, reading the data material to gain an overall impression, coding meaning units that represented experiences with the training performed and amalgamating the content from meaning units. In the last step, all authors worked together in an iterative analytical process.

## Results

### Feasibility

Participant characteristics are described in Table 1. The ability to perform the training varied among the participants. One participant withdrew before the pre-test because of time constraints and P9 withdrew due to a bursitis in the foot after seven training sessions (this participants outcome measures are not included). For the included participants that completed the study, the adherence to the exercises with guidance was 95% and 38% for home exercises. After pre-test P8 felt sore in the gluteus medius muscle and did not perform the first session strictly to the plan but had no trouble in the remaining sessions.

### Physical measures

Two participants (P6, P8) increased their eight-item HiMAT score and one participant (P2) decreased by more than two points. None of the participants had a clinically important change for the 6MWT (Table 2).

**Table 2:** 6MWT distance, heart rate and perceived exertion after testing and eight-item HiMAT

	6MWT						Eight-item HiMAT	
	Distance (m)		Heart rate (BPM)		Perceived exertion (Borg)		Score (pts)	
	Pre	Post	Pre	Post	Pre	Post	Pre	Post
P1	560	607	169	184	12	14	13	13
P2	505	495	124	121	9	9	13	10*
P3	600	582	150	157	11	11	10	11
P4	425	415	165	149	16	14	4	4
P5	491	500	195	194	14	19	12	11
P6	664	678	184	193	12	12	11	17*
P7	520	520	145	138	13	13	13	13
P8	642	661	177	175	15	17	11	17*

These were conducted at pre- and post-test

*Indicate clinical change between measurement points

*6MWT* six-minute walk test, *HiMAT* high-level mobility assessment tool

### Self-reported measures

Four of eight participants reported their walking function to be ‘much better’ at post-test, two ‘minimally better’ and two ‘unchanged’ (Table 3). The self-reported measure of perceived walking difficulties related to diagnosis was increased in four and decreased in three participants, and unchanged in one participant (Table 4). Three participants reported more fatigue at post-test, two reported less fatigue, two were unchanged and one had missing data (Table 4). Pain varied amongst measurement times. Pain scores equal to or above 3 were only reported by the oldest participant (P8) and those with bilateral CP (P4 and P5) (Table 4).

**Table 3:** Patient global impression of change compared to before intervention

Patient global impression of change				
	Week 2	Week 4	Week 6	Post
P1	Unchanged	Unchanged	Unchanged	Unchanged
P2	Minimally better	Much better	Much better	Much better
P3	Unchanged	Unchanged	Unchanged	Unchanged
P4	Unchanged	Minimally better	Minimally better	Minimally better
P5	Minimally better	Much better	Much better	Much better
P6	Minimally better	Much better	Much better	Much better
P7	Minimally better	Minimally better	Much better	Much better
P8	Minimally better	Much better	Much better	Much better

**Table 4:** NPRS during and at post-test and Walk-12 and FISSA scores measured at pre- and post-test

	NPRS				Walk-12	FISSA
	Pre	Week 3	Week 6	Post	Pre–post	Pre–post
P1	0	0	0	0	32–35	71–74
P2	2	0	1	1	20–32	31–41
P3	1	0	1	1	27–22	44–51
P4	5	0	7*	3*	68–68	110–111
P5	3	4	3	3	40–58	75–103
P6	0	1	0	1	33–28	64–45
P7	0	1	1	1	38–42	MI
P8	2,5	0	0*	5*	53–47	126–114

*MI* missing item, *NPRS* numeric pain rating scale, *FISSA* fatigue impact and severity self-assessment

*Indicate clinical change between measurement points

### Qualitative interviews

#### It was time-consuming to master the ballistic exercises and control the ankle

Ballistic training was new to all participants, and controlling the ankle joint in a limb with movement disorder was challenging. Training the affected side was experienced very differently for those with unilateral CP than training the unaffected side. For example, coordinating the calf muscle contraction and timing the movement was complicated. In the first part of the intervention, the active knee extensor muscles indicated incorrect exercise performance, which reminded them to reduce the compensating muscles and use the calf muscles more. This training demanded concentration. Sometimes they became tired and lost focus; hence, ballistic strength training was multi-tasking, controlling and strengthening the limb. Although, the number of repetitions forced their mind to control the motion in a completely new way. They shared a common frustration of not mastering the technique, reflecting upon ‘what am I actually doing in this exercise’ and how they planned the ankle motion attempting to activate it:

You need to try to connect it [the ankle flexors] and disconnect the others [the knee extensors], and how are you able to do that, right, it is this connection that is difficult because there is nothing natural to it…When I tell my right foot to do it, there is nothing automatic going on; I have kind of learned it in a way. (P8)

All participants reported that it was particularly difficult to find the rhythm of the exercise. However, after about four weeks of training they reported ‘mastering’ the exercise to a certain extent. Therefore, some provided feedback that more focus should be prioritized to perform the movement. The importance of having guidance from a trainer was the basis of perceived success. The glideboard did not challenge balance, which they felt gave more focus to the movement. When they mastered it in the final part of the later weeks, the training apparatus gave a particular rhythmic sound. This connection between muscles and rhythm of the exercise was described as hard to control. Therefore, they wished the study could be prolonged when the rhythmic sound became more automatic. Towards the end of the intervention, all reported greater awareness of the calf muscles during training. It had been a struggle, but with all the repetitions completed during the training session, they eventually got used to it:

I think there has been some progress that let me feel that I mastered it more at the end than in the beginning, but still, sometimes I got it and sometimes not…sometimes it's just right I think, and suddenly I can't do it again. (P1)

#### Various training responses and walking awareness

The participant's bodies responded differently throughout the period. On some days the leg muscles felt more tired than others, but they were unable to detect any pattern to how long the feeling lasted after the workout, whereas others did not feel anything at all. The knee extensors felt sorer in the first half of the intervention, explained by the long learning phase to master the exercises. They felt steadier during standing and walking with ballistic training and explained this as more muscle control, more focus on how the heels touched the ground and less stumbling when tired. One participant was able to jump after the intervention, something they had never managed before.

I feel a lot more support, you know a lot more, I have kind of more support around the ankle in the foot, so when walking I can feel that the foot isn’t that loose anymore, it became firmer, I am more determined in gait when walking. I notice. And I also feel that I have become much stronger. (P7)

Overall, they did not feel that walking speed had improved, but that their legs contributed more evenly to gait. They reported that the unaffected leg began to compensate less and the affected leg felt more involved. This raised hope that it was possible to achieve more balance between the sides. When the affected leg was in swing, they had to think ‘heel first, then toe’ before the foot hit the ground. Those with unilateral CP compared sides of the body, with one describing how the sound of the unaffected leg in a pedestrian tunnel helped control 'proper' heel-toe walking by matching the echo to the affected leg. The unaffected leg was considered dominant and they reported this side needed to be disconnected, and simultaneously the affected leg reconnected, to control push-off; something they found harder when tired. One participant expressed fascination on how little training was needed to improve walking and noticed a huge difference, by experiencing that the foot did not collapse during walking. In contrast, two participants described that they could not feel any changes due to the training. However, neither experienced their gait to be problematic. One described the conscious feeling of controlling the affected side:

I actually think more, yes, I have tried to walk, mmh, walk more properly then, and trying consciously to use the left foot like I think we are supposed to use the feet in a kind of way, yes, I actually try that, hoping this will be more automatic… (P2)

#### Addressing closer follow-up to home exercises

The participants experienced the ballistic exercises on the glideboard in the rehabilitation laboratory as a good supplement to their usual workout. However, all the participants found it hard to perform the home exercise targeting the hip muscles. Even though the exercises were available by video from a website, some found it hard to know if they had performed them correctly. Some used family members to help with a quality check. At least half conducted some of the home exercise sessions, even though not strictly to the plan. They called for closer follow-up when home exercises should be part of the training, especially when an exercise targeted muscles other than the calf muscles. One participant also suggested that the exercise could have been part of the ballistic warm-up routine to ensure it was done correctly. One described it like this:

It was exhausting, especially the running, or running in quotes, with hips high up, because that is a muscle I practically never needed. So, that was heavy, but I will continue doing that because I felt it gave results. (P6)

## Discussion

This study demonstrates that ballistic strength training is feasible for and well accepted by adults with CP. Both ballistic training and targeting the ankle were new to the participants, who reported a familiarization phase of about four weeks. There was no change in physical performance tested by 6MWT and HiMAT, although self-reported measures and findings from the qualitative interviews indicated otherwise.

### Evaluation of acceptability and suitability of intervention and study procedures

The most effective dose in ballistic training for people with CP is currently unknown [6]. With a different muscle morphology in spastic muscles [13], it is uncertain if the same recommendations as for healthy adults should be applied. Vershuren et al. [35] have published guidelines to increase muscle strength in CP. They suggest that once force has been developed in simple tasks more complex exercise activities could be added. In accordance with their suggestions, it is rationally to target the calf with ballistic strength training, before introducing functional power training in whole-body motions as suggested by Van Vulpen et al. [36]. Moreau et al. [10] suggested from their strength study with higher velocity movements in youth with CP that eight to 10 weeks and two to three times per week are effective at improving muscle power. Our study follows these recommendations for frequency and duration. The studies we found in the literature, including exercises performed with high velocity, are single-joint only, via an isokinetic dynamometer [10, 11, 37]. Our participants targeted the ankle plantarflexors by lying on a glideboard and simultaneously controlling the hip and knee beside the ankle push-off. They found it challenging to perform ballistic strength exercises and our study exercises might require greater motor control, since the motion was not facilitated by the equipment. Another plausible explanation for this might be an impaired RFD that has previously been found in the thigh [15] and ankle muscles [16]. These suggestions may explain why a long familiarization time was reported.

Coordination training targeting the ankle has been recommended to improve the walking pattern in children with CP [12]. Without coordination, the controlled application of force is compromised. Based on our qualitative results and the fact that the CP lesion is located in the brain [1], we argue that the participants also indirectly performed coordination training. For each laboratory session, three exercises with as many repetitions as possible within five minutes were performed (Additional file 1: Training log ). We could argue that this amount is more to increase local muscular endurance, but there is no established optimum for ballistic strength training. Rest periods are vital to ensure correct quality [9] and should have been included more structurally. Our participants did not have adequate control over the ankle and reported that all repetitions forced them to control the motion. When strength exercises set requirements for coordination, it is possible that basic motor control strategies should be integrated with basic strength training principles of progressive overload, specificity and variation [38] for a more holistic approach in this population. Intervention time is therefore an area for further exploration when targeting any muscle group. Our participants reported that exercises targeting the ankle plantarflexors were relevant, which was supported by the high adherence (95%) to the supervised laboratory exercises targeting the ankle. Our study has demonstrated that ballistic strength training targeting the ankle plantarflexor muscle is acceptable in a sample that never had trained the ankle plantarflexors ballistically before.

Adults with CP report more pain [39] and fatigue [40] than the general population. In this study, participants with bilateral CP and older participants reported higher pain levels [4]. Importantly, the perception of pain did not seem to increase during or after an eight-week ballistic training period. Three participants experienced a greater amount of fatigue at post-test, whereas two participants experienced less fatigue. From the interviews, some reported how periods with exams, or full-time and part-time work, led to an accumulated feeling of life becoming too hectic to proceed with the training intensity. An increase in total burden of activities to fit into a week potentially increased the FISSA scores for some participants. Factors affecting pain and fatigue should be considered in each individual prior to further training studies, including whether participants have enough time and capacity to complete the training intervention, although on their own they may not be barriers.

One participant withdrew from the study after seven training sessions because of a bursitis under the toes in the affected foot. The motion of the exercise was new to all participants; however, the load was less than body weight (from 15 to 60% of bodyweight). With an exploratory approach, we targeted the plantarflexors twice per week to account for enough restitution. Even though pain was monitored twice a week, this was not captured by the screening. It is possible that questions targeting the specific area could give better monitoring than just asking for pain in general. However, there is always a risk of injury in strength training and close monitoring is recommended.

### Evaluation and refinement of data collection procedures and outcome measures

In total, the participants attended 95% of the laboratory-based exercises. In these sessions the participants were monitored closely and guided with feedback, which was crucial to understanding the exercise criteria. This may explain the high adherence to the laboratory-based sessions. Experiences from the home exercises stated that they were cognitively challenging and were met with low (38%) adherence. Exercises to be performed at home should have followed the content in the laboratory-based exercises. Participants suggested this could be part of the warm-up routine of the laboratory-based sessions in the first weeks, to learn the motion before doing it independently.

After the ballistic intervention, participants’ perceived walking difficulties related to their diagnosis increased in P2 and P6 (Table 4), in contrast to walking ability that P2 rated as ‘much better’ after the intervention (Table 3). P2 also explained how walking became a more conscious activity when trying to walk better and use the affected side more. Even though these results seem conflicting, their perception might differ from the outcome measures, and both could be right.

Walking speed, walking endurance, gross motor function, spatiotemporal data, kinematics and kinetics have all been used as outcome measures to evaluate intervention efficacy in adults with CP [41, 42]. Walking is a complex motion that requires both RFD/power and motor control. Improvements in complex movements takes time and in our study, the eight-week intervention did not seem to produce a change in the desired outcome for walking (6MWT). We targeted ankle plantarflexors as the ‘bottle-neck’ of reduced forward propulsion and any change in muscle capacity should also have been investigated.

### Preliminary evaluation of participant responses to intervention

Six participants reported improvement in walking (PGIC), but the results from the walk test (6MWT) and gross motor function (HiMAT) did not confirm this perceived improvement. In a qualitative study, both intrinsic and extrinsic factors have been shown to influence daily walking in adults with CP [43]. The comprehension of the exercise is necessary for walking function and balance makes the perception that the exercise works important. Individual feedback on performance and progression was highlighted as crucial for progression by the participants. However, as training interventions for adults with CP have been few in Norway, this opportunity to receive training aiming to improve walking could trigger the placebo effect and might explain the discrepancy between the positive subjective reports and the lack of improvement in the objective measures.

A subjective feeling that the affected leg contributed more evenly to gait was reported from the interviews. We did not measure symmetry, and this might have improved with no change to walking speed. However, ballistic strength training can increase RFD, but it does not appear to have improved sufficiently to translate into improved 6MWT and HiMAT scores. Still, we believe that increasing the functional capacity for the muscles’ ability to generate force will also improve walking distance, but the motion must firstly be consolidated in a more prolonged intervention phase.

There is limited evidence in the literature supporting aerobic or strength training to improve motor function or increase gait speed on a group level in people with CP [6]. We have a small sample size with GMFCS I-II, but as a group, they are heterogeneous. With a small population and large variation, we need to properly understand the muscular physiological adaptation to ballistic strength training before including a more extensive study sample.

## Limitations

This study includes data on a small convenience sample and may not be representative of a general CP population. This study investigated only one of the three muscle groups responsible for propulsion during walking with ballistic strength training. Still, the calf muscles contribute the most in forwarding propulsion. Independent evaluators should have conducted the interviews, but we did not have the resources to employ them. The home exercises should have been closer monitored and controlled in order to gain effect. The reliability and validity of Walk-12 in CP are yet to be determined, but we used because it is a good measure for self-rated walking ability in other neurological conditions.

## Conclusion

This study demonstrated that ballistic strength training was feasible and suitable in adults with CP. However, guidance and a long (four weeks) familiarization time were reported necessary to master the exercises. Most participants reported self-experienced improvements, although no physical performance measures improved. Thus, prolonged intervention may be required for perceived physical improvements to emerge. Also, other outcome measures sensitive to power output remains to be investigated.

## Supplementary Information

**Additional file 1.** Interview guide, Training log, Home-based training and other training.

## Data Availability

The datasets used and/or analysed during the current study are available from the corresponding author on reasonable request.

## Abbreviations

CP: Cerebral palsy
6MWT: Six-minute walk test
HiMAT: High-level mobility assessment tool
PGIC: Patient global impression of change
NPRS: Numeric pain rating scale
FISSA: Fatigue impact and severity self-assessment
STC: Systematic text condensation
