# Supplementary material for: Can adults with cerebral palsy perform and benefit from ballistic strength training to improve walking outcomes? A mixed methods feasibility study
Source: BMC Sports Sci Med Rehabil. 2021 Dec 18;13:160. doi: 10.1186/s13102-021-00382-1 (PMC8684268; doi:10.1186/s13102-021-00382-1)
Supplement: Supplementary file 1 — Additional file 1. Interview guide, Training log, Home-based training and other training. [file 13102_2021_382_MOESM1_ESM.docx]

**Interview guide**

The purpose of this qualitative part of the study is to map your experience of having participated and whether participation has led to changes in daily life that we are unable to capture any other way.

The guide serves more as support for structure rather than a compulsory checklist for interviews.

- Short introduction to the interviewer and study background
- As you have read and signed, the interview is recorded but will be deleted
- You will be made anonymous in our further work with the interviews
- You can withdraw anytime during the interview without having to give any reason

Can you start by describing some specific experience that you have had as a participant during the intervention? Whether it is a positive or negative experience.

Based on your experiences of the supervised training program for the last eight weeks, what do you think of:

- The amount of training
- The training intensity
- The guidance
- How it was to learn these exercises (how long it took, what feedback you needed, what you did to succeed)
- Did the workout affect any of your daily activities?
- Do you feel your gait has changed, either way?

**Training log**

Registered mean incline on glideboard, sets and mean repetitions for each set during the sessions in the lab. Care should be taken with this overview, since counting reps and registering at the same time with guidance. In some sessions there were two instructors.

|  | **Participant 1** | | | | | | | | | **Participant 2** | | | | | | | | | **Participant 3** | | | | | | | | | **Participant 4** | | | | | | | | | | | |
| --- | --- | --- | --- | --- | --- | --- | --- | --- | --- | --- | --- | --- | --- | --- | --- | --- | --- | --- | --- | --- | --- | --- | --- | --- | --- | --- | --- | --- | --- | --- | --- | --- | --- | --- | --- | --- | --- | --- | --- |
|  | Jump squats | | | Single leg jumping | | | Jogging | | | Jump squats | | | Single leg jumping | | | Jogging | | | Jump squats | | | Single leg jumping | | | Jogging | | | Jump squats | | | Single leg jumping (R) | | | Single leg jumping (L) | | | Jogging | | |
|  | Inc | Set | Rep | Inc | Set | Rep | Inc | Set | Rep | Inc | Set | Rep | Inc | Set | Rep | Inc | Set | Rep | Inc | Set | Rep | Inc | Set | Rep | Inc | Set | Rep | Inc | Set | Rep | Inc | Set | Rep | Inc | Set | Rep | Inc | Set | Rep |
| S1 | 7 | 3 | 20 | NR | NR | NR | NR | NR | NR | 8 | 1 | 92 | 10 | 1 | 139 | 4 | 1 | 99 | 8 | 4 | 59 | 4 | 9 | 35 | 4 | 4 | 52 | 11 | 4 | 22 | 8 | 3 | 49 | 9 | 5 | 34 | NR | NR | NR |
| S2 | 8 | 3 | 23 | 8 | 3 | 31 | 7 | 3 | 122 | 9 | 4 | 36 | 9 | 3 | 45 | 9 | 6 | 59 | 12 | 4 | 35 | 11 | 6 | 31 | 14 | 5 | 66 | 11 | 5 | 34 | 8 | 4 | 43 | 8 | 4 | 65 | 13 | 6 | 53 |
| S3 | 11 | 6 | 27 | 9 | 6 | 38 | NR | NR | NR | 10 | 5 | 44 | 8 | 6 | 35 | 9 | 4 | 76 | 13 | 5 | 39 | 10 | 5 | 43 | 14 | 6 | 56 | 11 | 5 | 29 | 8 | 2 | 162 | 8 | 4 | 71 | 14 | 5 | 94 |
| S4 | 12 | 7 | 21 | 9 | 1 | 40 | 12 | 4 | 100 | 11 | 3 | 90 | 8 | 5 | 39 | NP | NP | NP | 13 | 4 | 55 | 10 | 5 | 51 | 14 | 4 | 101 | 13 | 4 | 60 | 8 | 3 | 99 | 8 | 3 | 100 | 14 | 4 | 134 |
| S5 | 12 | 6 | 25 | 9 | 5 | 68 | 12 | 4 | 100 | 11 | 3 | 57 | 8 | 5 | 34 | 9 | 6 | 32 | 13 | 4 | 38 | 11 | 5 | 50 | NR | 7 | 54 | 13 | 4 | 50 | 9 | 4 | 69 | 8 | 5 | 46 | 15 | 4 | 105 |
| S6 | 12 | 4 | 63 | 7 | 3 | 90 | 12 | 6 | 65 | 11 | 5 | 32 | 8 | 6 | 42 | 9 | 5 | 46 | 13 | 5 | 42 | 11 | 7 | 33 | 14 | 6 | 58 | 13 | 4 | 50 | 9 | 4 | 65 | 7 | 5 | 71 | 15 | 6 | 75 |
| S7 | 12 | 4 | 51 | 6 | 3 | 128 | 12 | 3 | 170 | 11 | 3 | 52 | 8 | 4 | 50 | 11 | 3 | 86 | 14 | 4 | 49 | 11 | 7 | 25 | 14 | 6 | 57 | 13 | 3 | 83 | 7 | 4 | 62 | 7 | 4 | 91 | 14 | 2 | 219 |
| S8 | 12 | 4 | 48 | 8 | 4 | 60 | 12 | 5 | 100 | 11 | 3 | 78 | 8 | 3 | 91 | 11 | 4 | 76 | 14 | 2 | 100 | 11 | 5 | 51 | 14 | 5 | 59 | 13 | 4 | 52 | 6 | 3 | 74 | 6 | 4 | 58 | 12 | 5 | 79 |
| S9 | 12 | 4 | 57 | 6 | 5 | 82 | 10 | 2 | 238 | 11 | 2 | 142 | 10 | 3 | 131 | 11 | 3 | 144 | 14 | 4 | 55 | 11 | 4 | 58 | 14 | 6 | 51 | 13 | 4 | 56 | 6 | 3 | 98 | 6 | 3 | 74 | 13 | 2 | 241 |
| S10 | 13 | 3 | 73 | 4 | 6 | 59 | 10 | 3 | 132 | 11 | 4 | 51 | 8 | 5 | 44 | 11 | 5 | 66 | 14 | 4 | 57 | 10 | 4 | 62 | 14 | 5 | 74 | 13 | 2 | 132 | 5 | 3 | 99 | 5 | 3 | 80 | 13 | 2 | 234 |
| S11 | 12 | 4 | 59 | 5 | 4 | 101 | 10 | 2 | 133 | 12 | 3 | 91 | 8 | 2 | 159 | 11 | 2 | 216 | 14 | 4 | 50 | 10 | 2 | 149 | 14 | 3 | 125 | 13 | 2 | 117 | 5 | 2 | 141 | 5 | 6 | 44 | 12 | 3 | 140 |
| S12 | 12 | 4 | 53 | 5 | 6 | 46 | 9 | 5 | 50 | 12 | 4 | 66 | 8 | 2 | 151 | 11 | 4 | 108 | 14 | 4 | 50 | 10 | 5 | 50 | 14 | 4 | 65 | 13 | 5 | 39 | 5 | 3 | 78 | 5 | 4 | 50 | 11 | 4 | 79 |
| S13 | 10 | 6 | 42 | 7 | 5 | 81 | 9 | 1 | 240 | 12 | 3 | 97 | 9 | 3 | 100 | 11 | 3 | 144 | 14 | 4 | 44 | 10 | 5 | 44 | 14 | 5 | 52 | 13 | 5 | 46 | 5 | 2 | 159 | 5 | 2 | 166 | 11 | 3 | 141 |
| S14 | 11 | 3 | 83 | 6 | 5 | 71 | 9 | 3 | 122 | 12 | 5 | 67 | 8 | 3 | 91 | 11 | 4 | 126 | 14 | 5 | 48 | 10 | 4 | 69 | 14 | 4 | 90 | 13 | 4 | 55 | 5 | 4 | 70 | 5 | 4 | 57 | 11 | 3 | 118 |
| S15 | 12 | 3 | 67 | 5 | 4 | 77 | 10 | 5 | 54 | 12 | 4 | 60 | 8 | 5 | 49 | 11 | 4 | 95 | 14 | 4 | 48 | 10 | 5 | 54 | 14 | 6 | 54 | NP | NP | NP | 5 | 4 | 54 | 5 | 3 | 50 | 9 | 3 | 143 |
| S16 | - | - | - | - | - | - | - | - | - | - | - | - | - | - | - | - | - | - | 14 | 2 | 150 | 10 | 4 | 76 | 14 | 4 | 109 | - | - | - | - | - | - | - | - | - | - | - | - |
|  | **Participant 5** | | | | | | | | | | | | **Participant 6** | | | | | | | | | **Participant 7** | | | | | | | | | **Participant 8** | | | | | | | | |
|  | Jump squats | | | Single leg jumping (R) | | | Single leg jumping (L) | | | Jogging | | | Jump squats | | | Single leg jumping | | | Jogging | | | Jump squats | | | Single leg jumping | | | Jogging | | | Jump squats | | | Single leg jumping | | | Jogging | | |
|  | Inc | Set | Rep | Inc | Set | Rep | Inc | Set | Rep | Inc | Set | Rep | Inc | Set | Rep | Inc | Set | Rep | Inc | Set | Rep | Inc | Set | Rep | Inc | Set | Rep | Inc | Set | Rep | Inc | Set | Rep | Inc | Set | Rep | Inc | Set | Rep |
| S1 | 7 | 3 | 30 | 6 | 5 | 40 | 8 | 4 | 55 | 6 | 5 | 41 | 7 | 5 | 22 | 6 | 4 | 43 | 6 | 2 | 50 | 12 | 5 | 22 | 8 | 4 | 48 | NR | NR | NR | NP | NP | NP | NP | NP | NP | NP | NP | NP |
| S2 | 10 | 6 | 21 | 7 | 6 | 31 | - | - | - | 10 | 4 | 53 | 9 | 5 | 20 | 7 | 6 | 34 | 11 | 5 | 52 | 13 | 4 | 36 | 10 | 4 | 56 | 14 | 4 | 56 | 9 | 4 | 43 | 6 | 5 | 91 | 11 | 4 | 108 |
| S3 | 9 | 5 | 17 | 7 | 4 | 41 | 7 | 5 | 38 | 10 | 3 | 64 | 9 | 5 | 42 | 7 | 4 | 51 | 12 | 4 | 66 | 10 | 4 | 50 | 11 | 4 | 63 | 15 | 4 | 91 | 10 | 4 | 62 | 5 | 4 | 49 | 11 | 3 | 195 |
| S4 | 11 | 3 | 63 | 8 | 4 | 56 | 6 | 5 | 34 | 11 | 2 | 156 | 9 | 3 | 93 | 7 | 3 | 95 | 13 | 3 | 144 | 15 | 6 | 28 | 11 | 2 | 125 | 13 | 4 | 85 | 11 | 3 | 77 | 6 | 4 | 67 | 12 | 3 | 132 |
| S5 | 12 | 4 | 35 | 8 | 5 | 39 | - | - | - | 11 | 3 | 87 | 9 | 6 | 25 | 7 | 4 | 63 | 13 | 4 | 83 | 16 | 5 | 39 | 11 | 4 | 63 | 13 | 5 | 64 | 11 | 6 | 25 | NR | NR | NR | NP | NP | NP |
| S6 | 12 | 4 | 35 | 8 | 5 | 45 | - | - | - | 11 | 4 | 50 | 10 | 5 | 28 | 7 | 4 | 61 | 13 | 3 | 117 | 16 | 4 | 50 | 11 | 5 | 50 | 13 | 7 | 47 | 11 | 5 | 44 | 6 | 4 | 63 | 12 | 6 | 53 |
| S7 | 12 | 4 | 46 | 8 | 4 | 75 | - | - | - | 11 | 3 | 97 | 10 | 3 | 60 | 7 | 3 | 110 | 13 | 2 | 188 | 16 | 4 | 50 | 11 | 5 | 49 | 13 | 7 | 46 | 11 | 2 | 124 | 6 | 1 | 348 | 12 | 3 | 135 |
| S8 | 12 | 4 | 45 | 12 | 4 | 62 | - | - | - | 11 | 4 | 63 | 11 | 4 | 38 | 7 | 4 | 60 | 13 | 4 | 85 | 16 | 4 | 42 | 11 | 4 | 70 | 13 | 4 | 78 | 11 | 3 | 77 | 5 | 3 | 103 | 7 | 4 | 70 |
| S9 | 12 | 3 | 66 | 8 | 2 | 173 | - | - | - | 11 | 3 | 91 | 10 | 3 | 66 | 8 | 2 | 171 | 13 | 2 | 213 | 15 | 4 | 51 | 11 | 2 | 156 | 13 | 3 | 127 | 11 | 3 | 90 | 4 | 3 | 94 | 7 | 3 | 144 |
| S10 | 12 | 2 | 118 | 8 | 4 | 72 | - | - | - | 11 | 2 | 150 | 11 | 4 | 43 | 8 | 5 | 60 | 13 | 4 | 83 | 15 | 4 | 76 | 11 | 2 | 179 | 13 | 3 | 119 | 11 | 4 | 60 | 4 | 5 | 47 | 7 | 4 | 100 |
| S11 | 12 | 3 | 80 | 7 | 5 | 47 | 5 | 5 | 39 | 11 | 4 | 75 | 12 | 3 | 73 | 8 | 1 | 364 | 14 | 2 | 185 | 15 | 3 | 89 | 12 | 2 | 151 | 13 | 3 | 124 | 11 | 2 | 136 | 4 | 1 | 311 | 7 | 3 | 158 |
| S12 | 12 | 3 | 80 | 7 | 2 | 157 | 5 | 3 | 53 | 11 | 3 | 100 | 12 | 3 | 73 | 8 | 2 | 149 | 15 | 2 | 190 | 15 | 4 | 50 | 13 | 5 | 47 | 13 | 6 | 45 | 9 | 4 | 63 | 5 | 3 | 90 | 7 | 5 | 68 |
| S13 | NP | NP | NP | 7 | 2 | 155 | 5 | 4 | 46 | 11 | 3 | 100 | 13 | 3 | 74 | 9 | 4 | 73 | 15 | 1 | 400 | 15 | 4 | 42 | 13 | 4 | 50 | 13 | 5 | 53 | 8 | 2 | 112 | 5 | 2 | 155 | 7 | 3 | 147 |
| S14 | - | - | - | - | - | - | - | - | - | - | - | - | 13 | 2 | 123 | 9 | 3 | 95 | 15 | 1 | 424 | 15 | 4 | 50 | 11 | 5 | 50 | 13 | 4 | 50 | 7 | 2 | 100 | 5 | 2 | 151 | 7 | 3 | 123 |
| S15 | - | - | - | - | - | - | - | - | - | - | - | - | 14 | 4 | 50 | NR | 4 | 55 | 16 | 1 | 428 | 15 | 4 | 41 | 11 | 5 | 46 | 13 | 5 | 54 | 7 | 3 | 80 | 5 | 3 | 100 | 8 | 4 | 90 |
| S16 | - | - | - | - | - | - | - | - | - | - | - | - | 14 | 3 | 95 | 10 | 4 | 96 | 16 | 1 | 431 | 15 | 5 | 51 | 11 | 5 | 58 | 13 | 5 | 64 | 8 | 2 | 122 | 5 | 2 | 176 | 8 | 3 | 176 |

NP = not performed, NR = not reported.

**Home-based training and other training**

The participants reported other training when they came to the laboratory sessions. Fitness class includes bootcamp, Zumba, basisball, step/strength etc.

|  | **Participant1** | | **Participant2** | |
| --- | --- | --- | --- | --- |
|  | Home-based training | Other training | Home-based training | Other training |
| w1 |  | None |  | 2 x Fitness class |
| w2 |  | Walk |  | Indoor rowing + strength |
| w3 |  | Bike + 16 km mountain hike |  | 2 x Fitness class |
| w4 |  | None |  | 3 x Fitness class |
| w5 | None | None | Home-based training | 3 sessions |
| w6 | Home-based training | None | Home-based training | 2 x Fitness class |
| w7 | Home-based training | None | Home-based training | Swimming + fitness class |
| w8 | None | None | Home-based training in water | Swimming |
|  | **Participant3** | | **Participant5** | |
|  | Home-based training | Other training | Home-based training | Other training |
| w1 |  | Treadmill running |  | None |
| w2 |  | 40 min indoor rowing |  | None |
| w3 |  | 2 x run + 3h mountain hike |  | None |
| w4 |  | 40 min ellipse/strength, 55 min run, 1,5 h skiing |  | None |
| w5 | None | Walk | None | Pool rehab |
| w6 | None | Ellipse | None | None |
| w7 | None | 1,5 h run | None | Pool rehab |
| w8 | None | Mountain hiking + 1 h run | Home-based training in water | Pool rehab |
|  | **Participant6** | | **Participant7** | |
|  | Home-based training | Other training | Home-based training | Other training |
| w1 |  | Physio |  | MI |
| w2 |  | None |  | 2 x run + 2 x mountain hike |
| w3 |  | Physio |  | 2 workout sessions |
| w4 |  | 2h walk + Walk |  | Run + Fitness class |
| w5 | 4 x Home-based training | None | None | None |
| w6 | 4 x Home-based training | None | None | None |
| w7 | None | None | 2 x Home-based training | None |
| w8 | None | None | Home-based training | Run + Mountain hike |
|  | **Participant9** | | **Participant10** | |
|  | Home-based training | Other training | Home-based training | Other training |
| w1 |  | None |  | Walk |
| w2 |  | None |  | 2 x 1,5 h mountain hike |
| w3 |  | 1,5 h mountain hike |  | None |
| w4 |  | Walk + mountainike |  | None |
| w5 | None | Swimming + Fitness class | None | None |
| w6 | None | 3 x Fitness class | None | None |
| w7 | Home-based training | Swimming + 2 x Fitness class | None | Mountain hike |
| w8 | Home-based training | Mountain hike + 2 x Fitness class | None | None |

MI = missing item
